# ‘They need to ask me first’. Community engagement with low‐income citizens. A realist qualitative case‐study

**DOI:** 10.1111/hex.13415

**Published:** 2022-01-15

**Authors:** Esther De Weger, Caroline Baan, Cheryl Bos, Katrien Luijkx, Hanneke Drewes

**Affiliations:** ^1^ Department of Quality of Care and Health Economics, Centre for Nutrition, Prevent and Health Services National Institute for Health and the Environment (RIVM) Bilthoven The Netherlands; ^2^ Department of Tranzo, Tilburg School of Social and Behavioural Sciences Tilburg University Tilburg The Netherlands; ^3^ Ministry of Health Welfare and Sports The Hague The Netherlands

**Keywords:** citizen involvement, community engagement, disadvantaged populations, low‐income, realist evaluation

## Abstract

**Background:**

Community engagement is seen as key to citizen‐centred and sustainable healthcare systems as involving citizens in the designing, implementation and improvement of services and policies is thought to tailor these more closely to communities’ own needs and experiences. Organizations have struggled to reach out to and involve disadvantaged citizens. This paper examines how if, why, and when low‐income citizens wish to be involved.

**Methods:**

For this qualitative realist case‐study, 19 interviews (one dyad) were held with (20) low‐income citizens in two Dutch municipalities. Additionally, the results were discussed with a reference panel consisting of professionals and citizens to enrich the results and to ensure the results had face validity.

**Results:**

The results showed four different ways in which low‐income citizens wished to be involved: (a) in a practical/volunteer way; (b) as a buddy; (c) as a lay expert; (d) not involved at all. The factors affecting citizens’ interest and capacity to participate include citizens’ own experiences of the services they access and their personal situations, e.g. their mental or physical health, extent of financial crisis, family situation, home environment. None of the interviewees was currently involved, but all had ideas for improving health(care) services and policies. Citizens’ experiences of the services they accessed acted as a motivator for some to be involved as they wanted to ensure others would not have the same struggles, while for others their own needs and an apathetic system remained too high a barrier. To enable involvement, citizens need continued support for their own health(care) and financial situation, better communication and accessibility from services, practical support (e.g., training and bus passes) and recognition for their input (e.g., monetary compensation).

**Conclusion:**

The study shows that citizens’ experiences of the services they accessed influenced if and how they wanted to be involved with health and care services. Despite the fact that all participants had shared solid ideas for improving services and policies, they were hindered by a bureaucratic, impersonal and inaccessible system. Organizations seem to underestimate the required investments to reach out to low‐income citizens and the support required to ensure their involvement.

**Patient and Public Involvement (PPI) in Study:**

Citizens as well as PPI organizations were members of the reference panel who helped formulate the research questions and recruitment strategy. The local reference panel also helped to interpret and refine the initial findings.

## 1 INTRODUCTION

Over the past few decades community engagement (CE) is increasingly seen as key to the development of citizen‐centred and sustainable healthcare systems, to the improvement of citizens’ health and well‐being, and to reducing health inequalities.^1^, ^2^, ^3^, ^4^ The aim of CE is to involve citizens in the decision‐making, planning, designing, governance and/or delivery of services and policies.^2^, ^5^ CE approaches can range from consultation—whereby citizens have limited power to influence decision‐making—to partnership and (shared) leadership—where citizens have decision‐making control.^1^, ^5^, ^6^ CE approaches can therefore take many different forms including citizen advisory panels, (one‐on‐one) peer healthcare delivery and informal care and support, or community‐led initiatives.^2^, ^7^ Despite the critical role CE is expected to play in the improvement of communities’ well‐being and in the quality of services and policies and despite the corresponding investments many organizations are placing within CE, important criticisms remain. One of the most important criticisms concerns the disparities in participation rates and that only a select group of citizens are enabled and empowered to participate, that is white, middle‐class and often retired citizens.^2^, ^3^ Especially young people and low‐income citizens go unrepresented in CE initiatives.^8^

Previous studies have shown that social inequities in engagement stem from the fact that CE approaches are mostly based on the interests, needs and norms of those designing the approaches and that ‘engagement environments’ are often built for efficiency in terms of time, money and tighter budgets, instead of citizen empowerment.^9^ Such emphasizing of efficiency over empowerment in engagement approaches results in a loss of influence for citizens, especially for those who are socioeconomically disadvantaged.^9^, ^10^, ^11^, ^12^ As such, when vulnerable or disadvantaged citizens are engaged, they report feeling shut out and unable to share their experiences and perceptions, their skills and insights—for example, because of poorly timed or advertised engagement activities, a lack of safe and trusting environments or power imbalances.^9^, ^11^, ^12^ This, in turn, can lead to citizens feeling frustrated, cynical and apathetic.^7^, ^9^, ^13^, ^14^, ^15^, ^16^, ^17^, ^18^, ^19^, ^20^, ^21^ Partly because only a select group of citizens are involved in the shaping, decision‐making, planning and/or governance of health and care services on behalf of whole neighbourhoods or communities, many CE approaches may fail to improve the health outcomes for all citizens and may thus even lead to a deepening of health inequality rates.^3^, ^22^, ^23^ Such criticisms are important to consider as without better inclusivity and diversity in the representation of CE approaches, it may be harder to ensure health systems and communities are reflective of diverse experiences and thus to address a wide variety of needs.^7^, ^9^

The previous literature has not just highlighted the structural issues with engagement approaches (i.e., organizations’ inaccessible structures and processes) but also indicated key areas for improvement. For example,^7^, ^14^ developed nine guiding principles for the successful implementation of CE approaches highlighting the importance of, for example providing supportive leadership, fostering safe and trusting environments, acknowledging and addressing power imbalances between citizens and organizations, ensuring citizens’ early involvement and creating a shared vision for CE. While, Cyril et al.^3^ pointed out key components that can specifically improve disadvantaged groups’ ability to be involved, including bidirectional learning and using peer‐delivery. Previous studies have also suggested the importance of properly investing in organizational cultures based on levelling power dynamics, giving voice to that previously excluded and true community empowerment, for example, and importantly by skilling‐up professionals in community outreach, engagement and empowerment.^3^, ^7^, ^9^

Despite previous studies highlighting how and why many 'mainstream' CE approaches exclude disadvantaged citizens,^7^, ^9^, ^13^, ^15^, ^17^, ^18^, ^20^, ^21^ organisations still struggle to design and implement CE approaches that either specifically *engage* disadvantaged groups or which are inclusive of a wider range of citizens.^7^, ^9^, ^22^ This is because many studies have highlighted where organizations have gone wrong, for example inaccessible organizational structures and processes, power imbalances, lack of safe and trusting environments,^7^, ^14^ but few have investigated what disadvantaged groups’ (support) needs, interests and motivations are to become involved.^2^, ^24^, ^25^, ^26^

The aim of this study was to examine how low‐income citizens’ experiences of health(care) and income‐support services influences if, when, how and why they wished to be involved in improving health(care) and income support services and policies. As such, this case‐study investigated low‐income citizens’ perceptions and experiences of the services they access and their interests in being involved with organizations to improve health(care) and support services and policies to ensure. This paper presents the results of a realist qualitative case‐study, which explored if, why, when and how low‐income citizens and citizens with financial support needs wish to be involved in helping others to improve their financial security and well‐being. The study's aim was to investigate the support low‐income citizens required to be involved and what the contextual factors and mechanisms were explaining their involvement preferences and needs.

## 2 METHODS

This case‐study is part of a 4‐year realist qualitative multiple case‐study evaluating the development of CE approaches in six different regions in the Netherlands. The multiple case‐study was undertaken in consultation with a reference panel. The panel consisted of stakeholders involved in developing CE approaches within the six different regions, including policymakers, involved citizens, patient and public involvement (PPI) organizations and experts in the field of public health, health inequalities and citizen participation (see Table 1). The panel therefore helped to ensure that the study addressed stakeholders’ questions regarding CE and addressed relevant gaps in the literature. The study was informed by the realist evaluation (RE) approach.

**Table 1:** Reference panel participant description

No.	Type of organization	Type of function
1.	Community‐led initiative	Volunteer, community‐led initiative board member
2.	Community‐led initiative	Volunteer, community‐led initiative board member
3.	Community‐led initiative	Volunteer village key worker, community‐led initiative board member
4.	Patient and public involvement organization	Representative role, outreach role
5.	Patient and public involvement organization	Representative role, project management role
6.	Patient and public involvement organization	Representative role, educational role for both citizens and organizations
7.	Patient and public involvement organization	Representative role, policymaker
8.	Municipality	Policymaker
9.	Municipality	Policymaker
10.	Municipality	Policymaker
11.	Municipality	Policymaker
12.	Health and care organization	Public health professional
13.	Knowledge institutes	Researcher
14.	Knowledge institutes	Researcher
15.	Knowledge institutes	Researcher
16.	Knowledge institutes	Researcher
17.	Knowledge institutes	Commissioner of research

RE is especially suitable for complex topics and interventions. To understand how an approach might generate different outcomes under different circumstances, RE uses context‐mechanism‐outcome configurations (CMOs) to unearth the underlying changes in reasoning and behaviour of participants that are triggered within particular contexts (or by particular contextual factors). More so than other methods, RE seeks to answer: ‘what works, how, why, for whom, to what extent and under which circumstances’.^27^, ^28^ In this way, RE helps to not just study people's experiences but helps to understand the underlying causation for those experiences.^29^

By using RE the study sought to understand the causation behind low‐income citizens’ involvement preferences and support needs with the expectation that this would enable organizations to better understand citizens and thus tailor their involvement approaches and support to citizens’ needs and experiences (see Table 2 for definitions for realist concepts).

**Table 2:** CE‐oriented definitions of realist concepts^7^, ^14^, ^28^, ^30^

Intervention	Refers to interventions’ implemented activities, strategies and resources,^31^ e.g.,: citizen advisory panel meetings, or neighbourhood organized workshops.
Context	Pertains to the backdrop of a programme and examples of context include, e.g., pre‐existing processes, cultural norms and history of an organization or community in which a programme is implemented, geographic location effects, funding sources, opportunities or constraints. Contexts can therefore be understood as any condition that triggers or modifies the behaviour of a mechanism.
Mechanism	Mechanisms describe how the resources embedded within a programme influence the reasoning and behaviour of programme participants. Mechanisms are usually hidden, sensitive to variations in context and generate outcomes, e.g., citizens feeling more empowered due to learning opportunities.
Outcome	Refers to intended, unintended, or unexpected programme outcomes on the micro, meso or macro level, e.g., sustainability, quality and integration of services *(macro)*, citizens’ level of involvement in health and care services (e.g., in designing policies) *(meso)*, citizens’ health and well‐being outcomes *(micro)*.
CMO	CMO is a heuristic used to explain generative causation. CMOs help to reflect on the relationship between a context, mechanism and outcome of interest in a particular programme. CMOs can be about a whole programme or only certain aspects of a programme. Formulating and refining CMOs is largely how researchers analyse data in RE as it allows for a deeper understanding of which (aspects of) interventions work, for whom, under which circumstances and to what extent.^30^ CMOs are also used to generate or refine programme theories, which, in turn, help shape the final product of an evaluation (e.g., recommendations). CMOs are also to generate or refine programme theories.
Programme theories	Is a hypothesis about how a programme or programme component may or may not work, under what contexts and with what outcomes. In this study, the guiding principles (De Weger et al.^14^, ^29^), which can be seen as action‐oriented programme theories, were used as initial programme theories.

Abbreviations: CE, community engagement; CMOs, context‐mechanism‐outcome configurations.

### 2.1 Recruitment strategy and study sample

For this case‐study, the authors collaborated with two Dutch municipalities and a PPI organization that wanted to better understand low‐income citizens’ financial support and involvement needs. Municipality A wanted to improve their work and income policy by aligning it better to citizens’ needs and experiences and was chosen as their developmental questions aligned with the study's objectives. Municipality B was chosen on the advice of the PPI organization. According to the PPI organization, municipality B had developed the best‐practice low‐income support services within the region. The researchers hoped that the differing contextual factors within the municipalities would help to compare and contrast low‐income citizens’ experiences accordingly (see Table 3).

**Table 3:** Municipalities description

Municipality A	*Facts about the municipality*: rural municipality with favourable unemployment and welfare support rates. The national unemployment average is 3.8% and in the municipality the unemployment rate is 2.7%. The national welfare support rate is 5.2% and in the municipality it is 1.9%.^32^ *Reason for including municipality*: municipality wanted to improve their employment and income policies and support services by aligning them better to citizens’ needs and experiences.
Municipality B	*Facts about the municipality*: rural municipality with unfavourable unemployment and welfare support rates. The national unemployment rate is 3.8%, and in the municipality, it is 5.1%. The national welfare support rate is 5.2%, and in the municipality, it is 6.2%.^32^ *Reason for including municipality*: municipality with, according to the involved PPI organization, best‐practice unemployment and income support services

Abbreviation: PPI, Patient and public involvement.

Acknowledging that the recruitment of a ‘hard‐to‐reach’ group would take more time and effort, the researchers had prioritized the additional efforts it might take and not set any specific timeframes for the recruitment, focussing instead on achieving data saturation and ensuring all those who wanted to participate were given the chance to do so in the manner that suited them and their needs. For this study, purposive and snowball sampling^33^ was used to recruit citizens who were living in the two municipalities and were, for example, long‐term unemployed, employed but receiving a (too) low income and were therefore living under or around the local poverty line, in significant and long‐term debt, receiving or entitled to receive (income) support for their financial instability. Firstly, all citizens receiving welfare support from the municipalities were sent an invitation letter—approximately 200 letters were sent out and eight participants had responded to the letter. Secondly, the municipalities’ support workers reached out to clients who were receiving income support and they felt were stable enough and interested in sharing their story (purposive sampling). Thirdly, citizens were also approached by the researchers themselves through local food banks (researchers went to the local food banks themselves and collaborated with the volunteers), nonprofit emergency funds (who like the support workers were asked to reach out to clients who were on income‐support and they felt would be interested in taking part in an interview), and local churches (who researchers collaborated with to invite anyone who was on income‐support and interested in taking part in the study (purposive sampling). Approaching citizens through their support workers had the highest response rate (12). When potential participants reached out to the researchers about the interview, researchers discussed with potential participants that they were under no obligation to take part in the interviews and that (non)‐participation would have no impact on any of the welfare support they were receiving. The interviews were held when, where and how citizens would like to meet to reduce the burden on participants and to foster a safe and trusting environment. Because the interviews had taken place during the COVID‐19 pandemic, participants could choose whether they wanted to conduct the interviews over the telephone, via video calls, face‐to‐face in the municipalities’ ‘corona‐proof’ rooms, or face‐to‐face at participants’ own homes, to ensure participants felt as safe and comfortable as possible. To help stimulate interest, to induce participants to participate, and to show our appreciation for the participants taking the time and effort to share their stories with us, we provided €35 gift cards redeemable at the majority of shops and supermarkets. The recruitment and outreach took 5 months (May–September 2020) and ultimately 21 interviewees were recruited for this study—with one interview being a dyad. This dyad interview was a couple who were refugees. Both wanted to share their experiences but only one spoke English and thus provided their own answers and provided translations for the other person.

### 2.2 Data collection

To aid data analysis, all interviews were recorded and transcribed. All study participants had received an information letter and had provided their informed consent. A semistructured topic guide was used to ensure the same questions were discussed during each interview in an open and nonstructured way (see Supporting Information Appendix I). The interview findings were anonymized and aggregated, after which the summarized findings were presented to the reference panel to ensure findings had face validity and rigour. Data saturation had been achieved by interview 14 and 19 interviews had been conducted as information *(when no new themes emerged and when there was a high rates of recurrence of response)*. Furthermore, additional interviews and already been planned and *were therefore held as conformation and to* provide participants with the opportunity to share their stories as well.^34^ Ultimately, 19 interviews (one of which was a dyad interview) lasting between 1 and 2 h were held with a total of four respondents from municipality A and seventeen respondents from municipality B. Five interviews were conducted over the telephone and fourteen interviews were held face‐to‐face interviews. Interestingly no participant chose to do video call interviews (e.g., through Zoom, Whatsapp or FaceTime). The majority of interviews were conducted in Dutch; however, three interviews were conducted in English. The study received ethics approval from the corresponding university (reference EC‐2017.96) and the interviews were held between September and October 2020.

### 2.3 Data analysis

This study took an inductive and deductive approach for analysing the data, using previous literature and theories on CE, poverty and welfare support to form the basis of the analysis approach. This study is part of a 4‐year multiple case study that examined how CE was being developed and implemented in six different regions in the Netherlands. The first stage of the study consisted of an international rapid realist review regarding the barriers and enablers for engaging communities and developed eight guiding principles for the successful implementation of CE. The second stage of the study confirmed and refined the guiding principles: (a) ensure staff provide supportive and facilitative leadership to citizens; (b) foster a safe and trusting environment enabling citizens to provide input; (c) ensure citizens’ early involvement; (d) share decision‐making and governance control with citizens; (e) acknowledge and address citizens’ experiences of power imbalances between citizens and professionals; (f) invest in citizens who feel they lack the skills and confidence to engage; (g) create quick and tangible wins; (h) take into account both citizens’ and organizations’ motivations; (i) develop a shared vision with clear roles for professionals and citizens, ensuring communities’ diversity is reflected within the vision.^7^, ^14^ Building on from the previous stages of the study, this study used the guiding principles as initial programme theories and thus helped to inform the analytical framework. This means that previous literature and theories on CE, poverty and welfare support helped to inform the interview questions, coding tree and overall analysis but that during the analysis there was space for new themes and insights to emerge (see COREQ checklist for additional information).

Furthermore, to examine how citizens’ perceptions and experiences of the support services they access influence if, why, when and how they would want to be involved with municipalities and health(care) organizations to improve other citizens’ financial security and overall well‐being, the authors constructed CMOs within each transcript. This helped the authors’ understanding of the contextual factors and mechanisms underlying citizens’ involvement preferences and their support needs enabling their involvement. The interviews were thus coded and analysed using CMOs, which were then drafted and analysed in MaxQDA by E. D. W., refined and confirmed by H. W. D. and C. B. and finally discussed by all authors. To aid the authors during the data analysis process and to ensure consistency and transparency, the authors applied the same CE‐oriented definitions of ‘interventions’, ‘contexts’, ‘mechanisms’ and ‘outcomes’ (see Table 2). Furthermore, the MaxQDA coding process was based on previous literature and models on the social determinants of health and poverty (see Supporting Information Appendix I and II).^35^, ^36^, ^37^, ^38^ Additionally, the nine guiding principles for successful CE were used as the theoretical framework to examine the underlying contextual factors and mechanisms explaining citizens’ involvement preferences.^7^, ^14^ The clustering followed a deductive and inductive, sequential and iterative process, which has been applied in previous studies and described elsewhere^7^, ^14^:

(1) CMOs were coded and clustered into citizens’ support needs, their preferred way in helping other citizens improve their financial security and overall well‐being.
(2) The authors discussed the clusters and thematically analysed, reviewed and discussed them again.
(3) The final draft of the clustered CMOs was shared with all authors to confirm and refine the themes.
(4) After the final draft of the CMOs, the findings were presented to the reference panel. The panel discussed the validity, relevance and applicability of the findings within their own local contexts, confirming that the results had face validity.

## 3 RESULTS

The following section will first describe how citizens’ perceptions and experiences of the support services they access influence their involvement preferences, including their reasons for wanting to be involved (or not) and their preferred methods of involvement. Secondly, the support citizens would require from municipalities and other health(care) organizations to be successfully involved in ensuring they and others in their communities are financially secure will be examined. Throughout this section, examples of CMOs will underpin the results and further CMO examples can be found in the Supporting Information Appendix III.

### 3.1 Interview participants

The 21 interviewees had a wide variety of backgrounds, employment and educational experiences, family situations and support needs. Many interviewees had become unemployed due to physical health issues (e.g., musculoskeletal, degenerative diseases) and/or mental health issues (e.g., post traumatic stress disorder, autism), others due to family breakdowns or domestic violence, some due to a history of drug abuse, others were on income‐support as they had come to the Netherlands as refugees and were trying to build a new life in the country. Many interviewees were struggling with a complex combination of these issues. The vast majority of interviewees had negative experiences of the support services they accessed and described services as uninterested and unsympathetic and/or as fragmented and bureaucratic. As the section below will show these personal contextual factors and the organizational/systematic contextual factors influenced whether they wanted to be involved or not, but also negatively impacted their ability to do so.

### 3.2 Citizens’ involvement preferences (Tables 4 and 5)

**Table 4:** Summary of participant characteristics, experiences, involvement preferences and required support for involvement

	Practical involvement	Buddy involvement	Lay expert	No involvement
Who	Functioning with mental health issues, signed off from work, complex family situations	More stabilized mental health, high debt, difficulty finding work	‘Only’ complex financial/debt situation, unable to find work	Own needs too high, experiences with services too negative
Experiences/why	Meaningful activities, wanting to contribute	Negative experiences, and wanting to prevent similar experiences for others	Convinced policies and services can be improved	‐ Still in crisis, not feeling their situation is stable enough ‐ Negative experiences led to distrust
Required support	Boundaries are respected	Boundaries are respected	Investment in skills and knowledge	Unaware of their skills/ability and their useful ideas
	*Organizational/service contextual factors that need to be improved to enable involvement*: Empathy, person‐centred approach, transparency, accessibility			

**Table 5:** Description of study participants

No.	Basic participant demographics	Employment history	Involvement/volunteer experience
1.	Female, Dutch, aged 25–30	None	None
2.	Female, Dutch, aged 40–55	Intermittent periods of employment and intermittent periods of unemployment	None
3.	Female, Dutch, aged 40–55	Long history of employment, currently unemployed due to health reasons and family breakdown	None
4.	Female, Dutch, aged 55–65	Long history of employment but due to health issues and family breakdown now long‐term unemployed	Some experience of lobbying municipality for wheelchair access
5.	Male, Dutch, aged 55–65	Intermittent periods of employment and intermittent periods of unemployment	None
6.	Female, Dutch, aged 40–55	Long history of employment, but after being laid off, currently long‐term unemployed	None
7.	Female, non‐Dutch and basic Dutch, aged 40–55	No formal history of employment but lots of unpaid labour due to domestic abuse	None
8.	Male, aged 40–55	Some history of employment, but sporadic due to addiction issues	None
9.	Male, refugee, no Dutch, aged 50–65	History of employment in the country of origin. No employment in the municipality due to refugee status	None
10.	Female, refugee, average Dutch, aged 50–65	History of employment in the country of origin and some employment in the municipality. Currently unemployed	None
11.	Male, Dutch, aged 18–25	No paid employment history due to mental health	Part time, unpaid work as part of mental healthcare plan
12	Male, Dutch, aged 50–65	Long history of (self)‐employment. Now unemployed due to bankruptcy and family breakdown	None
13.	Female, refugee, no Dutch, aged 40–55	No history of formal employment in municipality due to refugee status	None
14.	Male, Dutch, aged 40–55	Long history of employment. Recently employed again	None
15.	Male, Dutch, aged 50–65	Intermittent periods of employment and intermittent periods of unemployment, due to mental health issues. Recently employed part‐time again	None
16.	Female, Dutch aged 50–65	History of employment but due to health issues and family breakdown, now long‐term unemployed	None
17.	Male, refugee, no Dutch, aged 20–35	No history of employment in municipality due to refugee status	None
18.	Male, Dutch, aged 20–35	Intermittent periods of employment and intermittent periods of unemployment due to addiction issues. Currently unemployed	None
19.	Female, Dutch, aged 20–35	Some employment history, but due to mental health issues and family breakdowns, currently unemployed	None
20.	Female, Dutch, aged 30–45	Intermittent periods of employment and intermittent periods of unemployment due to family breakdown. Currently unemployed	None
21.	Male, Dutch, aged 50–65	Long history of employment but due to health issues and family breakdown now long‐term unemployed	None

The results indicate that different contextual factors and mechanisms influence if, how and why citizens want to be involved in improving other citizens’ financial situation and underlying social determinants of health. The most important factors that affect citizens’ interest or capacity to participate include citizens’ own experiences of the services and their own personal situations (e.g., their mental or physical health, seriousness of their financial situation, complexity of family situation). Broadly speaking, the results indicate that there are four different ways in which low‐income citizens want to be involved with organizations: (a) in a practical/volunteer way; (b) as a buddy; (c) as a lay expert; (d) no involvement at all (see Table 6). It is important to note that there was overlap between the four categories in that some interviewees wished to be involved, for example as a buddy and a lay expert or would have liked to have been involved as a lay expert but currently felt unable to as they were still too much in crisis. In the following section, this paper will further explain the contextual factors and mechanisms underlying the four types of involvement preferences (see Supporting Information Appendix III for more CMO examples and Table 5 for a summary of low‐income citizens according to health and care needs, experiences and involvement preferences).

**Table 6:** Description of involvement categories and underlying motivational factors

Practical	Buddy	Lay expert	No involvement
Volunteering not focused on people's personal stories or issues and is instead practical in nature	Volunteering focused on one‐to‐one interaction with individuals first accessing unemployment and/or debt services. Those wanting to be a buddy for others want to use their own long history of accessing services to help others	Volunteering focused on improving debt, income support and unemployment services. Such volunteering would require collaboration with organizations and professionals (instead of with people accessing services)	No volunteering, but may be interested in sharing their stories and experiences on a one‐time or sporadic basis (because they felt unable to invest their time and energy on a long‐term basis)
For example litter pick up, organizing meetings	For example providing listening ear, helping to fill out forms	For example providing service‐user perspective to improve policies and services, collaborating with professionals to provide or improve services	For example one time conversation
Citizens who wanted to be involved (regardless of their preferred involvement category) were motivated to do because they wanted to spend their time more meaningfully and to add some structure to their day‐to‐day routine There is overlap between the buddy and lay‐expert involvement categories due to the desire to improve services and policies for others going through the same issues. However, those preferring the buddy involvement category were focused on one‐to‐one interaction, while those preferring the lay‐expert category were more focused on collaborating with organizations and professionals. Those not wanting to be involved at all (beyond sharing their experiences on a one‐time basis) described not feeling stable enough to be involved on a long‐term basis.			

Three interviewees wanted to be involved *in a practical/volunteer* way as they wished to contribute to their own community in the hopes of building up their social contacts. Examples interviewees gave were for example organizing meetings between health(care) organizations and low‐income citizens, or picking up trash and cleaning up their neighbourhood's parks and gardens with others. One interviewee stated that she had experienced the care and financial support services as unaligned to her own needs and the processes and structures to apply for financial support as too complicated and fragmented *(context*). She wanted to contribute to the community and support others in financial difficulty but was worried that one‐on‐one support would become too personal and difficult to cope with (*mechanism*). Thus she would prefer to be involved in a practical, more organizational manner (e.g., organizing meetings between citizens and the municipality (*outcome*). However, she stated that she would need organizations to reach out to her and ask her to become involved and to provide clear boundaries for her involvement (*outcome*).

If they're looking for people to help organise things, then I'm open to it, because I'm someone who's always willing to fight for other people. But supporting people one‐on‐one…that's…that would be too difficult for me. I'm highly sensitive, so I would end up taking other people's stuff home and that would be way too much for me…so if there's anything organisational I can do, something more abstract to help with…. (Municipality A, female participant 2)

A larger number of interviewees (five) wanted to be involved as a ‘buddy’. As a buddy they wished to help other citizens find their way through the bureaucratic process of trying to receive help for their financial situation and/or unemployment, for exampel by finding the right forms to fill in or the right contact persons within health and care organizations or to lend a listening ear. For example, one interviewee described how she had experienced the system as inaccessible (*context*) and that through her own experiences she fully understands how stressful unexpected high costs can be for people in debt (*context*). She feels it is important to contribute to society and sees it as her calling to support people who have newly entered financial and debt relief services (*mechanism*). She wants to support the newcomers with the knowledge she has gained over years of accessing these services herself (*outcome*).

For someone receiving income‐support, who is in debt, I think a buddy who sometimes listens to you is of great value. (Municipality A, female participant 20)

A slightly larger number of interviewees (six) wanted to be involved as a *lay expert* to help improve the inaccessible, fragmented and unpersonal support services and organizational structures and processes, which low‐income citizens are confronted with. For example, one interviewee said that due to his very negative experiences of financial support and debt services he has a clear sense of how services and policies can be improved (*context*). He really wanted to be involved with the municipality to ensure people in poverty and in debt are truly listened to and heard. He wants to be involved because of his strong conviction that services and policies can and should be improved for others (*mechanism*). By sharing his own thoughts, he wants to ensure organizations develop more person‐centred services that are better aligned to people's holistic needs (*outcome*). Another interviewee mentioned how she had experienced the system as inaccessible and untransparent (*context*). Through her own personal experiences, she understands how stressful it is to be on income support and to be in debt (*context*). She also learned by doing her own digging that different municipalities provide different types of support (*context*). This motivated her to share her experiences and her research with her own municipality because she feels that there is nothing more important than to bring low‐income citizens’ struggles to light (*mechanism*). She hopes that through her involvement as a lay expert that municipalities and support organizations get a better view of what low‐income citizens truly need and how services and policies can be improved (*outcome*).

Because I'm wholly convinced that things can and should change. And I want to contribute in my own small way: listen guys, we're not friends, but give your opponents, your critics, let me prove that I'm right. That things can change and improve. Give people who are against the current poverty policies a chance to implement improvement projects. By simply listening. Just by simply listening to people who want to be involved to improve things (Municipality A, male participant 12)

Finally, a majority of people *could not or did not want to be involved* in any shape or form (11). The majority of interviewees within this group said they did not want to participate because they were afraid they would not be able to cope with their own situation. A smaller number of interviewees said they did not want to participate because they simply no longer trusted organizations due to their own negative and stressful experiences. Some interviewees who did not want to participate could see themselves being involved if they received the proper support and/or compensation while being involved and thus enabled to be involved in their own preferred way. For example, one interviewee stated that her support workers had advised her to do volunteer work to spend her days more meaningfully; however, she still struggles with her physical and mental health issues (*context*). Another interviewee, with a history of drug abuse and unemployment, mentioned he would in theory like to be involved as a buddy to help others with a history of drug abuse, but currently felt unable to focus on anyone else but himself and stabilizing his own situation. Another interviewee said she had had purely negative experiences with income‐support due to a lack of communication, lack of consistent support and bureaucratic mistakes (*context*). Because of these negative experiences she feels abandoned by the services meant to support her (*mechanism*). She is unmotivated to be involved with organizations to ensure others receive better care and support from services and only wants to focus on herself and her own family (*outcome*).

I think, yeah, I think that for me, I've had three really hard years on income‐support and with the debt services, and those experiences have cut me so deep, it still feels like a dark period, I don't feel any need to be involved and help others. I feel like I still need help myself. (Municipality A, female participant 4)

My support worker wanted me to do volunteer work…But my illness, that fluctuates, it's never the same…and actually I need to be mentally more stable first before I start a new challenge… (Municipality B, female participant 3)

### 3.3 Support citizens require to enable their involvement

First of all, it is worth noting that none of the interviewees were, at the time of interviewing, involved in any of the organizations or volunteer work. It could therefore be argued that by not providing the support low‐income citizens require to be involved, citizens miss out on chances to be involved and organizations miss out on ideas to improve their services and policies. The results indicate that improved accessibility and communication is vital to enable low‐income citizens’ involvement as this will help tackle the constraining systematic contextual factors facing citizens for their involvement. To start with, interviewees mentioned the need to be approached by organizations and to have their participation options explained. Interviewees also highlighted the importance of ongoing support for their own (mental)health, well‐being and financial status and that any potential involvement on their part would not put any more stress on these factors. Furthermore, interviewees discussed the importance of feeling heard and listened to by organizations and feeling valued and recognized for their (potential) contributions. Some interviewees mentioned that they would need training, for example to be a lay expert or buddy, for refugees in learning Dutch. Finally, some interviewees mentioned that (monetary) compensation for their involvement would also help them feel more motivated and to enable them practically to get involved (e.g., for transport costs) (see Supporting Information Appendix II for more CMO examples).

For example, one interviewee experienced that financial support services were very ‘business like’ and that the professionals had very little empathy for people presenting to the services in crisis (*context*). She really wanted to be involved as a lay expert to support people in crisis, to improve organizations’ communication to low‐income citizens and to ensure organizations took a more person‐centred approach, but she feared she would not be able to cope with the responsibility and added pressure (*mechanism*). To become a lay expert, she would need support for her own financial status and well‐being, she would need the training to help her fulfil the role of lay expert and mentioned a small monetary contribution (*outcome*). Only one interviewee had said that she had approached the municipality and told them she wanted to be involved to build up her social contacts and to help her spend her time meaningfully (*mechanism*). However, the municipality had never gotten back to her about the different ways she could get involved and is still waiting to hear back from them (*outcome*). This means the municipality missed the opportunity to involve citizens (*outcome*). All interviewees had shared useful ideas and suggestions for improving services and policies and for aligning these better to their own needs and lived experiences. This includes participants who had said that they would not or could not be involved because they were too much in crisis themselves, were distrustful of organizations or because they would need support from organizations first.

I need to be invited to have a conversation [with the municipality]. If the municipality were to open the door to me, that's easier and more motivating than having to ask to be let in. (Municipality A, male participant 18)

### 3.4 Panel deliberations

Both professionals and citizens within the panel highlighted that they found it a challenge to involve harder‐to‐reach groups. Professionals within the panel recognized that their communication and accessibility should be improved, while citizens highlighted that organizational communication and accessibility was also often a barrier to their own involvement. In an effort to improve accessibility, professionals recognized the need for them to develop trusting relationships with (low‐income) citizens. They thought that using a neutral partner or community figures could help them to build such relationships. They also discussed the compensation they could provide citizens for their involvement. Finally, based on these results, the professionals expressed a wish to develop a broader spectrum of involvement opportunities for citizens to try and align their CE approaches more closely with citizens’ interests and needs. However, professionals expressed that to improve their accessibility and to broaden citizens’ involvement opportunities, they would need to be given more resources and space to innovate their approaches.

## 4 DISCUSSION

Using the RE approach, this case‐study investigated if, how, why and when low‐income citizens wished to be involved with municipalities and health(care) organizations to improve health(care) and support organizations’ services and policies. The study shows citizens’ perceptions and experiences of the services they accessed, that is the underlying contextual factors and mechanisms, influenced their involvement preferences. The study indicates low‐income citizens can contribute to the improvement of health and care services and policies by sharing their ideas, needs and experiences as all interviewees shared ideas to improve services and policies. A majority of the interviewees did not want to participate at all due to personal and systematic/organizational contextual factors like mental and physical health or bureaucratic and inaccessible processes. For those willing to be involved, broadly speaking, the study identified three different categories: (a) in a practical/volunteer way; (b) as a buddy; (c) as a lay expert (see Table 6). However, none of the interviewees had been enabled or asked by organizations to get involved in any shape or form, further underscoring the systematic/organizational contextual issues that negatively impacted their ability to be involved. This highlights that by excluding low‐income citizens and preventing them from discussing their experiences and needs and the ways in which they want to be involved and the support they require to enable them to do so successfully and sustainably, organizations are missing out on important ideas to improve their services, policies and organizations. This suggests organizations also miss out on opportunities to ensure the health and care system is more inclusive and representative of a wider variety of citizen needs and experiences. On an important note, while neither the interviewees nor the organizations would describe participants sharing their experiences and ideas during the interviews as ‘involvement’, such activities could be seen as a first step to enable low‐income citizens to be involved in the manner best suited to their own interests and needs, thus enabling them to improve services and policies.

This study's interviewees’ negative experiences of apathy, bureaucracy, fragmentation and never being asked about their experiences or whether they would like to be involved shows the importance of tackling constraining systematic/organizational contextual factors.^3^, ^9^, ^14^ The lack of involvement and outreach from organizations towards low‐income citizens is in line with how citizens had experienced the services they had accessed: as unpersonal and apathetic, as bureaucratic and fragmented. Previous literature suggests that consecutive (western) national governments’ policies regarding benefits and income‐support have been focussed on improving efficiency and effectiveness. This has made it more difficult for organizations to deploy resources to alleviate poverty and social exclusion.^12^, ^39^, ^40^ *Previous authors like Cortis* ^12^ suggest that this is a major reason why organizations tend to over‐provide to those easiest to reach and assist and where results are more demonstrable and at the same time underprovide to more disenfranchised citizens who are more ‘challenging’ and costly to assist. Such a policy environment makes it more difficult for organizations to assist more marginalized groups and to promote more equal involvement.^39^, ^40^ The reference panel discussions underscored such findings as organizations had not seemed to prioritize the outreach of ‘harder‐to‐reach’ groups. They stated they were still searching for outreach and engagement activities more specifically tailored to disadvantaged groups’ needs.

This study highlights that organizations can improve their processes and structures by focussing on citizens’ own experiences and perceptions and that organizations should align their outreach and engagement strategies more closely to their lived experiences and needs.^3^, ^7^, ^14^, ^39^ The study highlighted the importance of reaching out to citizens on their own terms as all interviewees appreciated the opportunity to talk about their own experiences, perceptions and needs. It also showed that while it takes time and effort to reach out to harder‐to‐reach groups, like low‐income citizens, it may not be as hard as many organizations and professionals make it out to be. We, first of all, prioritized reaching out to low‐income citizens and decided to take all the time we needed to recruit and build relationships with low‐income citizens through those who are in closer contact with them, for example their support workers, local food banks, nonprofit emergency funds and local churches. Furthermore, we conducted the interviews, when, where and how low‐income citizens preferred highlighting that we prioritized their needs. For example, we adapted our interviews for refugee participants by conducting the interviews in English and by conducting a dyad‐interview to reduce the language barrier and to ensure participants felt more comfortable. While the language barrier may have affected the information we collected during the interview, we felt it more important to ensure refugees’ experiences and preferred involvement preferences were included in our data collection. Without making such adjustments we likely would not have been able to conduct the interviews at all. Our experiences underscore that organizations should work to make the outreach and engagement of low‐income citizens a priority and help citizens feel welcomed to be involved in a manner that suits them and their needs. Importantly, organizations should provide ongoing support to citizens for their own health, care and financial support needs and ensure that outreach and CE approaches are based on their own motivations and needs instead of health and care organizations’ interests. For example Roets et al.,^41^ highlighted the importance of supporting welfare recipients in dealing with administrative procedures, listing the needs of low‐income service‐users and improving the accessibility of organizations with aligned communication methods. This suggests that the guiding principles described in Section Results, 3, ^7^, ^14^ should be further refined to highlight that harder‐to‐reach groups, such as low‐income households, may need additional upfront support to enable their involvement in a manner that suits their ongoing complex health and care needs, for example through tailored outreach, specific support for their health and care needs, and leadership to tailor CE approaches to their specific motivations. Such upfront support may well differ from the support and investment already‐engaged citizens require (as highlighted within the original guiding principles*)*.

Finally, by examining citizens’ involvement preferences, this study highlighted the importance of examining and addressing citizens’ motivation to be involved.^7^, ^14^, ^42^ By investigating whether citizens are (a) ‘intrinsically motivated’—like the interviewees who wanted to do practical volunteer work to build their social networks or to have a daily routine—or are (b) ‘identified regulated’—like the interviewees who wanted to be involved as buddies or lay experts because they felt it was important to improve services and policies for others—and/or (c) ‘externally motivated’—for example like the interviewees who were motivated to take part in this study because of the gift vouchers—organizations can become more sensitive to citizens’ motivations and needs.^43^ It will also help organizations to understand when to provide rewards (e.g., training, stipends) to trigger ‘external motivation’ when involvement approaches are too far removed from citizens’ own personal motivating factors. Understanding citizens’ motivating factors is especially important when trying to reach out to harder‐to‐reach groups whose feelings of distrust towards (local) government and public sector services negatively impact their motivation to become involved.^44^

### 4.1 Study limitations

One limitation is the fact that this case‐study only included citizens from two rural municipalities. Presumably, the rural context of this case‐study influenced several important factors, including the availability and accessibility of health, care and financial support services available to interviewees. This limitation was mitigated by the reference panel's workshop discussions as this confirmed the validity and applicability of our interview findings in other contexts, thus further validating and enriching the interview findings.

### 4.2 Future studies

This case‐study seems to indicate that low‐income citizen involvement is possible if organizations were to prioritize reaching out to harder‐to‐reach groups based on citizens’ own needs and experiences, rather than on organizations’ interests. The study also suggests that low‐income citizens have valuable insight and suggestions for the improvement of services and policies. Future studies could investigate if and how low‐income citizens’ involvement would affect services and policies and ensure they are indeed better aligned with citizens’ needs and lived experiences.

## 5 CONCLUSION

This study investigated how low‐income citizens’ perceptions and experiences of the services they access influence if, why, when and how they wished to be involved with health and care services to ensure all citizens in their communities are financially secure. The study shows that citizens’ experiences of the services they accessed influenced if, why, when and how they wanted to be involved with health and care services. All citizens, whether they wanted to be involved as (a) in a practical/volunteer way; (b) a buddy; (c) lay expert or (d) not at all, all citizens had solid ideas for improving services and policies and aligning them better to citizens’ own needs and experiences, rather than organizations’ interests. However, despite these solid ideas for improving services and policies, participants were prevented from sharing these by a bureaucratic, impersonal and inaccessible system. Organizations should not underestimate the required investments to reach out to low‐income citizens and provide the required support to ensure their involvement (including e.g., person‐centred and empathetic approaches, transparency and accessibility, training, support for their complex needs). Such investments will help reduce the bureaucratic barriers citizens experience and will help them feel more listened to. With low‐income citizens’ involvement, organizations can improve their services and policies and ensure these are more aligned with all citizens’ needs.

## CONFLICT OF INTEREST

The authors have no completing interests or conflicts of interest.

## ETHICS STATEMENT

This study received ethics approval from Tilburg University (reference: EC‐2017.96). All participants were provided with information letters concerning the study and had time to ask any questions they may have had. It was also made clear that participation was completely voluntary. Afterwards, all participants signed forms stating their consent to participate. This is in accordance with Dutch national guidelines: https://www.tilburguniversity.edu/upload/ddc3ce11-1e82-4bf7-ac6d-e813999e5037_CODE%20OF%20ETHICS%20FOR%20RESEARCH%20IN%20THE%20SOCIAL%20AND%20BEHAVIOURAL%20SCIENCES%20DSW%20J%20%20%20.pdf and http://ec.europa.eu/research/participants/data/ref/fp7/89867/social-sciences-humanities_en.pdf.

## AUTHOR CONTRIBUTIONS

Research design was developed by Esther De Weger and reviewed and improved by Hanneke Drewes, Katrien Luijkx, Caroline Baan. Esther De Weger and the reference panel conducted the recruitment and Esther De Weger conducted the interviews. Esther De Weger, Hanneke Drewes and Cheryl Bos analysed and interpreted the data with feedback from Caroline Baan and Katrien Luijkx. Esther De Weger wrote the manuscript and Hanneke Drewes, Katrien Luijkx and Caroline Baan critically reviewed all drafts. All authors made substantial contributions to the conception and design and approved the final manuscript.

## Supporting information

Supporting information.

Supporting information.

Supporting information.

Supporting information.

## Data Availability

All data generated and analysed during this study are included in the published article and supplementary information files. Templates used for data extraction and analysis are available from the corresponding author on reasonable request.
