# Supplementary material for: ‘They need to ask me first’. Community engagement with low‐income citizens. A realist qualitative case‐study
Source: Health Expect. 2022 Jan 15;25(2):684–96. doi: 10.1111/hex.13415 (PMC8957733; doi:10.1111/hex.13415)
Supplement: Supplementary file 1 — Supporting information. [file HEX-25--s002.docx]

**Consolidated criteria for reporting qualitative studies (COREQ): 32-item checklist**

| **No. Item** | **Guide questions/description** | **Reported on Page #** |
| --- | --- | --- |
| **Domain 1: Research team and reﬂexivity** |  |  |
| *Personal Characteristics* |  |  |
| 1. Interviewer/facilitator | Which author/s conducted the inter view or focus group? | Methods, page 5 |
| 2. Credentials | What were the researcher’s credentials? E.g. PhD, MD | Title page (EdW and CB have an MSc. HD, KL and CaB have PhDs). |
| 3. Occupation | What was their occupation at the time of the study? | Title page (Researchers & Professors) |
| 4. Gender | Was the researcher male or female? | Not reported (female) |
| 5. Experience and training | What experience or training did the researcher have? | Not reported (interviewer has extensive experience conducting qualitative interviews and has been trained accordingly. Also has training working with vulnerable groups) |
| *Relationship with participants* |  |  |
| 6. Relationship established | Was a relationship established prior to study commencement? | Researchers took time to reach out to participants and to find out their interview preferences (e.g. time, location and language preferences). Prior to starting the interviews, researchers took the time to build rapport and make participants feel comfortable. |
| 7. Participant knowledge of the interviewer | What did the participants know about the researcher? e.g. personal goals, reasons for doing the research | Researchers shared the reasons and motivations for doing the research |
| 8. Interviewer characteristics | What characteristics were reported about the inter viewer/facilitator? e.g. Bias, assumptions, reasons and interests in the research topic | Researchers shared their reasons for the study and their interests in the research topic.  Interviewer bias and reflexivity are important to the validity of qualitative research. It is directed at the social interaction between the interviewer-interviewee, or what Kvale stated as the ‘asymetrical power relations of the researcher interviewer and the interviewed subject’.  We adjusted for such bias, and also ensured the rigour of our findings, through peer-briefing. Firstly, in addition to the first interviewer, two researchers checked the interview transcripts and investigated the codes also checking for bias. Secondly, through the reference panel we were able to again check for bias and validate the findings. |
| **Domain 2: study design** |  |  |
| *Theoretical framework* |  |  |
| 9. Methodological orientation and Theory | What methodological orientation was stated to underpin the study? e.g. grounded theory, discourse analysis, ethnography, phenomenology, content analysis | The realist approach was applied.  The aim of this study was to examine (parts) of this realist question: “if, why, when, and how low-income citizens wished to be involved, what support citizens required to be involved and what the contextual factors and mechanisms were explaining their preferences and support needs. These are inherently realist research questions (De Weger & van Vooren et al 2020). The researchers felt it important to understand the causation in their preferences and support needs as this would help organisations better understand and tailor their involvement approaches and support to citizens’ needs and experiences.  Through the use of CMO configurations, the realist approach enables researchers to unpack the causal pathways more than other research methodologies. |
| *Participant selection* |  |  |
| 10. Sampling | How were participants selected? e.g. purposive, convenience, consecutive, snowball | A description of sampling approach is provided in the methods section pages 4-5 |
| 11. Method of approach | How were participants approached? e.g. face-to-face, telephone, mail, email | A description of sampling approach is provided in the methods section pages 4-5. |
| 12. Sample size | How many participants were in the study? | A description of sampling size is provided in the methods section pages 4-5 |
| 13. Non-participation | How many people refused to participate or dropped out? Reasons? | A description of sampling is provided in the methods section pages 4-5 |
| *Setting* |  |  |
| 14. Setting of data collection | Where was the data collected? e.g. home, clinic, workplace | A data collection is provided in the methods section pages 4-5 |
| 15. Presence of non-participants | Was anyone else present besides the participants and researchers? | N.A. |
| 16. Description of sample | What are the important characteristics of the sample? e.g. demographic data, date | Table 3 provides basic demographic details of participants. Table 4 provides short description of the two municipalities. |
| *Data collection* |  |  |
| 17. Interview guide | Were questions, prompts, guides provided by the authors? Was it pilot tested? | The interview questions have been provided in Appendix III. The questions were run past the PPI organization as way of test. |
| 18. Repeat interviews | Were repeat interviews carried out? If yes, how many? | N.A. |
| 19. Audio/visual recording | Did the research use audio or visual recording to collect the data? | N.A. |
| 20. Field notes | Were ﬁeld notes made during and/or after the interview or focus group? | Fieldnotes were collected to enhance the iterative data collection process |
| 21. Duration | What was the duration of the interviews or focus group? | A description of this is stated in the methods section on page 5 |
| 22. Data saturation | Was data saturation discussed? | A description of data saturation is noted in the methods section on page 5 |
| 23. Transcripts returned | Were transcripts returned to participants for comment and/or correction? | N.A. With informed consent, interviews were transcribed verbatim |
| **Domain 3: analysis and ﬁndings** |  |  |
| *Data analysis* |  |  |
| 24. Number of data coders | How many data coders coded the data? | EdW was primary coder and CaB and HD checked, adjusted and refined the coding done. |
| 25. Description of the coding tree | Did authors provide a description of the coding tree? | Coding tree has been provided as an appendix. The coding tree had been developed based on the authors’ previous research on CE, based on the literature regarding CE with disadvantaged groups and based on the literature regarding poverty/low-income support/welfare. At the start of the study, the researchers conducted an evidence scan on poverty and low-income support to ensure the research design was informed by existing theories and previous literature. Prior to the study, we had conducted a rapid realist literature review regarding CE and conducted interviews and focus groups on the experiences and perceptions of CE. These previous studies also informed the research design.  We took a deductive and inductive approach meaning that our research questions and coding tree were informed by previous theories and literature, but was open to new insights. |
| 26. Derivation of themes | Were themes identiﬁed in advance or derived from the data? | In line with the RE approach, the study was conducted in an deductive & inductive (iterative) manner. This means that some themes/initial programme were identified in advance of the data collection and analysis. But that during the course of data collection and analysis, new themes were generated. This is discussed in the main manuscript in the methods section, in 5. |
| 27. Software | What software, if applicable, was used to manage the data? | MaxQDA, page 5 |
| 28. Participant checking | Did participants provide feedback on the ﬁndings? | Participants were provided the opportunity to reflect on and to provide feedback on the findings, but none had taken the opportunity to do so. |
| *Reporting* |  |  |
| 29. Quotations presented | Were participant quotations presented to illustrate the themes/ﬁndings? Was each quotation identiﬁed? e.g. participant number | Quotations are presented throughout the results section after each minor & major themes for transparency |
| 30. Data and ﬁndings consistent | Was there consistency between the data presented and the ﬁndings? | Yes, there is consistency, which was tested and confirmed within the research team and the local reference panel. This is discussed in the manuscript in the results and discussion sections |
| 31. Clarity of major themes | Were major themes clearly presented in the ﬁndings? | Yes, results were clearly presented and this was confirmed by our internal reference panel. |
| 32. Clarity of minor themes | Is there a description of diverse cases or discussion of minor themes? | Yes, results were clearly presented and this was confirmed by our internal reference panel. |
