# Supplementary material for: ‘They need to ask me first’. Community engagement with low‐income citizens. A realist qualitative case‐study
Source: Health Expect. 2022 Jan 15;25(2):684–96. doi: 10.1111/hex.13415 (PMC8957733; doi:10.1111/hex.13415)
Supplement: Supplementary file 2 — Supporting information. [file HEX-25--s001.docx]

**Appendix I_Summary of interview questions (translated from Dutch)**

**Theme 1: What would help you to make ends meet**

1. What helps you to make ends meet?

- Probing questions on basis of visualization of what the literature highlighted were the most important factors impacting financial situation (e.g. housing, employment, education, access to white goods, support of friends & family, mental and physical health)

1. What problems do you experience that prevent you from making ends meet?

**Theme 2: how can the municipality help you to make ends meet**

1. Have you ever asked the municipality or other services for financial support?

- If they did access services: what went well, what didn’t go well? Why?
- What could be improved?
- If they did not access services: why not? What prevented you from accessing services?
- How could the municipality, other services improve their services?

1. What prevents you from accessing services for income, employment support?

- To what extent do organisations/services have a good understanding of your needs? Of your situation?
- What can organisations do to improve this?

**Theme 3: how would you like to be involved with the municipality and/or other organisations to help others to make ends meet**

1. How would you like to be involved with the municipality to prevent poverty and/or to make others make ends meet

- Do organisations/municipality understand what’s necessary to prevent poverty? Why?
- Do you think organisations/municipality listen to you?
- What projects, activities would you be most interested in to participate in?
- How would you like to be involved: probing questions based on visualization of what the literature highlighted were important factors/ways for citizens to be involved?
- Why? What would help you to be involved?
- What support would you need to be involved? Why?
