# Supplementary material for: ‘They need to ask me first’. Community engagement with low‐income citizens. A realist qualitative case‐study
Source: Health Expect. 2022 Jan 15;25(2):684–96. doi: 10.1111/hex.13415 (PMC8957733; doi:10.1111/hex.13415)
Supplement: Supplementary file 3 — Supporting information. [file HEX-25--s003.docx]

**Appendix II: Summary of coding tree (translated from Dutch)** 7,14,36,38,39

1. **Stated definition of CE**
2. **Support needs**

- Type of support required/enablers & barriers for involvement
- Reasons support is required
- Other/additional

1. **Type of involvement**

- Lay expert
- Peer-delivery
- Sharing needs of target group with organisations
- No involvement
- No clear idea of what involvement would look like
- Other/additional

1. **Motivation for (non)involvement**

- To add some structure and meaning to day-to-day life
- To improve policies, services for others
- To improve communication, accessibility of services for others
- To highlight the stark reality of being in debt, negative experiences of debt & income support services
- Other/additional

1. **Social determinants of health**

- Physical and mental health
- Education
- Income and financial resources
- Support from friends & family (social safety net)
- Employment and employment opportunity in community
- Health and care services in community
- Living environment (e.g. housing, public transport)
- Cultural background
- Other/additional

1. **Experiences & perceptions of health and support services**

- Accessibility
- Person-centred
- Stigmatisation
- Consistency in support workers
- Collaboration between services (healthcare, municipal, income/debt support, etc)
- Other/additional

1. **Quotes**
