# Supplementary material for: ‘They need to ask me first’. Community engagement with low‐income citizens. A realist qualitative case‐study
Source: Health Expect. 2022 Jan 15;25(2):684–96. doi: 10.1111/hex.13415 (PMC8957733; doi:10.1111/hex.13415)
Supplement: Supplementary file 4 — Supporting information. [file HEX-25--s004.docx]

**Appendix III: Summary of CMOs underpinning themes**

| **Citizens’ involvement preferences** | | | | | | |
| --- | --- | --- | --- | --- | --- | --- |
|  | **Context** | | **Mechanism** | | **Outcome** | |
| 1. | - Currently has no job, no social network due to move to a new neighbourhood - History of substance misuse and homelessness | | He feels he no longer contributes to the community, because he no longer does any (un)paid work. He also misses the routine that (un)paid work provides | | He would like to volunteer doing practical jobs in the community (like picking up trash & tidying up the parks) so he can contribute in some small way & to have a sense of structure | |
| 2. | - She had very negative experiences of financial support services which were fragmented and bureaucratic and unpersonal. - There was very little coordination between the support services | | Because she understands what it is like to be in a long-term crisis situation, she feels motivated to be involved with the municipality & support services for other low-income citizens | | She wants to improve the communication & accessibility between low-income citizens & organisations to ensure organisations do not just work ‘according to the book’ and to develop a person-centred approach to financial services | |
| 4. | - She had very negative experiences of financial support services which were fragmented, bureaucratic and unpersonal. - There was very little coordination between the support services - She has experienced how stressful it is to be in financial crisis | | Because of a sense of community and with the interest of society in mind, she wants to understand and improve the processes and structures of municipal financial services | | - Through her involvement she wants to hold the municipality accountable. - She also wants to support others in a financial crisis and to activate them to get involved too - But in order to be involved, the municipality needs to be open to (low-income) citizens’ involvement | |
| 6. | - High functioning autism - Finds the processes & structures to apply for financial support too bureaucratic and difficult to navigate. Often citizens do not know what support is available or how to apply for the support | | In theory, she would like to be a lay expert to help the other citizens navigate the processes and structures, but is worried it would become too emotional/therapeutic which she feels would be beyond her capacity | | She would first need to be stable enough herself in order to be involved as a lay expert | |
| 7. | High functioning autism diagnosis and has experienced her care & support services as unaligned to her own needs, interests and capacity | | Fact that the care she receives is unaligned makes that she does not feel like she is working to her full potential | | This means she currently feels unable to contribute to the community, but is looking for ways to meaningfully pass the time | |
| 8. | She has had very negative experiences of the financial support services and has received very inconsistent ‘support’ | | Because of these negative experiences, she feels angry and does not have the energy to help others or be involved with organisations | | She only wants to focus on herself and her own family | |
| 9. | - She experienced that the care and support services hardly communicate or think about what her own personal needs are - Occupational therapists are currently investigating whether she is able to return to work or not (from a physical health perspective) | | She wants to be involved in her own way which suits her current capacity, because she wants to build up her social contacts and find some meaning in her day-to-day life again | | However, organisations do not communicate about the possible ways in which she could be involved/contribute to the community. This means she is still waiting | |
| 10. | - Interviewee had history of unemployment and drug abuse. - He had had negative experiences of financial support services and mental health services and experienced the lack of a lay expert or buddy supporting him through the recovery process | | He would want to become a buddy or lay expert himself, but feels like his situation is too unstable and currently incapable to participate | | He therefore cannot be involved as a lay expert or buddy | |
| **Support citizens require to enable their involvement** | | | | | | |
|  | | **Context** | | **Mechanism** | | **Outcome** |
| 1. | | He would like to volunteer doing practical jobs in the community (like picking up trash & tidying up the parks) so he can contribute in some small way | | Currently he goes out and picks up litter & tidies up local parks on his own, but lacks a sense of acknowledgement and practical support from the municipality | | - He would like the municipality to provide practical support (e.g. tools) - He would like the municipality to help form a small group of volunteers to enable social contact between volunteers |
| 2. | | - High functioning autism & ADHD - Currently on an unpaid work placement 5-6 hours a week, but is frequently asked to work more hours | | Because the organisation where he works, nor the municipality, provide him any sort of compensation, he feels demotivated | | Without the compensation he feels underappreciated and feels like his hard work is ignored |
| 3. | | - Unemployed and negative experiences of care and financial support services - In theory he would like to support other low-income citizens as a buddy | | He feels motivated to prevent others from having the same negative experiences of financial support services and would like to start contributing to the community again | | However, he would need support (e.g. debriefs with a coach) to ensure he does not take anyone’s emotional problems home with him |
| 4. | | Experienced the financial support services and the organisational processes and structures as very negative and frustrating | | Due to his very negative experiences he feels very motivated to share his ideas for improvement so policies & services are better aligned to people’s needs and experiences and therefore more person-centred | | But he would need the municipality to be open & willing to criticism and would need to actively listen to his improvement ideas. |
| 5. | | She’s experienced the financial support services as very unpersonal and unsympathetic | | In theory, she would like to be a lay expert to ensure the municipality and support services show more empathy towards people in crisis. However, she is worried she would currently not be able to cope with the added pressure and responsibility | | To be involved as a lay expert, she would need support herself, e.g. in the form of monetary compensation, be provided with training |
| 6. | | - She has had very negative experiences of mental health care services and financial support services. - She has PTSD and requires an intensive mental health care treatment. However, the organisation is unwilling to provide her this intensive treatment as long as she does not have child care support for her dependents | | In theory she would like to be a lay expert to improve the communication between organisations and between organisations and citizens, however without the mental health care for her PTSD and child care support, she feels unable to be involved | | With suitable mental health care support she would be able to start being involved and maybe even to find a job eventually |
| 7. | | - Interviewee is feeling more stable because he has been clean for several years and has suitable housing and able and willing to be involved in a practical way - However, because he is more settled he no longer receives any support anymore | | Because he no longer has any contact with his support workers or with the others in the programme, he feels lonely and somewhat abandoned. | | What he really needed is occasional contact with a support worker highlighting what (un)paid jobs or involvement options would be available to him so he can start building a new social network |
